# Arginase II activity regulates cytosolic Ca^2+^ level in a p32-dependent manner that contributes to Ca^2+^-dependent vasoconstriction in native low-density lipoprotein-stimulated vascular smooth muscle cells

**DOI:** 10.1038/s12276-019-0262-y

**Published:** 2019-06-03

**Authors:** Bon-hyeock Koo, Dongeui Hong, Hyeon Don Hong, Hyun Kyo Lim, Kwang Lae Hoe, Moo-Ho Won, Young Myeong Kim, Dan E. Berkowitz, Sungwoo Ryoo

**Affiliations:** 1 Department of Biology, School of Medicine, Kangwon National University, Chuncheon, 24341 South Korea; 2 Department of Anesthesiology and Pain Medicine, Yonsei University Wonju College of Medicine, Wonju, 26426 South Korea; 3 Department of New Drug Discovery and Development, Chungnam National University, Daejeon, 34134 South Korea; 4 College of Natural Sciences and Neurobiology, School of Medicine, Kangwon National University, Chuncheon, 24341 South Korea; 5 Departments of Molecular and Cellular Biochemistry, School of Medicine, Kangwon National University, Chuncheon, 24341 South Korea; 6 Department of Anesthesiology and Critical Care Medicine, Johns Hopkins Medical Institutions, Baltimore, MD 21287 USA

**Keywords:** Cardiovascular diseases, Vascular diseases

## Abstract

Although arginase II (ArgII) is abundant in mitochondria, Ca^2+^-accumulating organelles, the relationship between ArgII activity and Ca^2+^ translocation into mitochondria and the regulation of cytosolic Ca^2+^ signaling are completely unknown. We investigated the effects of ArgII activity on mitochondrial Ca^2+^ uptake through mitochondrial p32 protein (p32m) and on CaMKII-dependent vascular smooth muscle cell (VSMC) contraction. Native low-density lipoprotein stimulation induced an increase in [Ca^2+^]m as measured by CoCl_2_-quenched calcein-AM fluorescence, which was prevented by Arg inhibition in hAoSMCs and reduced in mAoSMCs from ArgII^−/−^ mice. Conversely, [Ca^2+^]c analyzed with Fluo-4 AM was increased by Arg inhibition and ArgII gene knockout. The increased [Ca^2+^]c resulted in CaMKII and MLC 20 phosphorylation, which was associated with enhanced vasoconstriction activity to phenylephrine (PE) in the vascular tension assay. Cy5-tagged siRNA against mitochondrial p32 mRNA (sip32m) abolished mitochondrial Ca^2+^ uptake and induced activation of CaMKII. Spermine, a polyamine, induced mitochondrial Ca^2+^ uptake and dephosphorylation of CaMKII and was completely inhibited by sip32m incubation. In mAoSMCs from ApoE-null mice fed a high-cholesterol diet (ApoE^−/−^ +HCD), Arg activity was increased, and spermine concentration was higher than that of wild-type mice. Furthermore, [Ca^2+^]m and p32m levels were elevated, and CaMKII phosphorylation was reduced in mAoSMCs from ApoE^−/−^ +HCD. In vascular tension experiments, an attenuated response to vasoconstrictors in de-endothelialized aorta from ApoE^−/−^ +HCD was recovered by incubation of sip32m. ArgII activity-dependent production of spermine augments Ca^2+^ transition from the cytosol to the mitochondria in a p32m-dependent manner and regulates CaMKII-dependent constriction in VSMCs.

## Vascular disease: Controlling arterial constriction

Researchers have illuminated how a protein, arginase II (ArgII), is involved in development of vascular diseases such as atherosclerosis, or narrowing of the arteries by plaque deposits. Blood vessel diameter is regulated by layers of muscle; the balance between constriction and relaxation is critical for blood flow and vascular health. Increased ArgII is known to be a factor in arterial disease; however, the details of regulation, and how they relate to plaque deposition, remain poorly understood. Sungwoo Ryoo at Kangwon National University, Chuncheon, South Korea and co-workers investigated how ArgII levels affect arterial constriction and relaxation in mice. Decreasing ArgII restored the muscle cells’ contraction response by preventing excessive calcium accumulation in the cellular powerhouse, mitochondria. These results may aid in developing treatments for one of the leading causes of death worldwide.

## Introduction

Arginase hydrolyzes L-arginine to urea and L-ornithine, a precursor for polyamines, including spermine, spermidine, and putrescine. Two isoforms of arginase are found in a variety of cells and may be expressed in species- and tissue-specific manners^1–5^. Arginase I (ArgI) is found in the cytosol, and arginase II (ArgII) is targeted to the mitochondria. Although ArgI can stimulate proliferation through a polyamine-dependent mechanism in aortic smooth muscle cells^6^, the mechanism underlying the role of ArgII in mitochondrial function is yet unknown. We previously demonstrated that ArgII played an important role in the regulation of mitochondrial Ca^2+^ concentration in native low-density lipoprotein (nLDL)-treated hAoSMCs, although we did not define the target protein^7^. Together with the endoplasmic reticulum, the mitochondria play a critical role as a Ca^2+^-handling organelle in cells, and mitochondrial Ca^2+^ influx from the cytosol affects cellular physiology and pathophysiology through contributions to the spatial and temporal profiles of intracellular Ca^2+^ signaling.

Calmodulin contains four EF-hand Ca^2+^ binding sites and is a major Ca^2+^ sensor and regulatory protein. In vascular smooth muscle cells (VSMCs), increased cytosolic concentration of Ca^2+^ ([Ca^2+^]c) activates the multifunctional Ca^2+^/calmodulin-dependent kinase II (CaMKII)^8^. The activated CaMKII provokes vascular smooth muscle cell (VSMC) contraction through interactions with myosin light chain kinase (MLCK), leading to MLC phosphorylation and actin-myosin interaction^9,10^. Furthermore, increased levels of intracellular Ca^2+^ may function as a second messenger to activate other signaling molecules^11–13^.

Initially, p32 was characterized as a pre-mRNA splicing factor SF-2 associated protein^14^, as hyaluronan-binding protein 1 (HABP1), as receptor for globular head domains complement 1q (gC1qR), or as complement 1q binding protein (C1qbp)^15^. Moreover, p32 is a conserved eukaryotic protein that exists in multiple subcellular compartments^16–18^. However, recent studies have demonstrated that p32 is primarily targeted to the mitochondria because the 73 N-terminal amino acids contain a mitochondrial localization sequence^19^. Mitochondrial p32 protein has been studied previously in cancer development in the context of mitochondrion morphology^20^, the maintenance of oxidative phosphorylation^21^, and ARF-induced apoptosis^22^. However, the primary function of p32 in the mitochondrion is yet unknown.

Although arginase activity in VSMCs provokes cell proliferation in a polyamine-dependent manner^6^, the protein regulated by arginase activity and its function in the mitochondrion are not yet known. To determine the physiological contribution of ArgII activity to vasoconstriction in VSMCs from humans and mice, we investigated how ArgII activity modulates [Ca^2+^]c and [Ca^2+^]m through p32. Furthermore, we tested the underlying mechanism of p32-dependent vasoconstriction in de-endothelialized vessels.

## Materials and methods

### Materials

We purchased 2(S)-amino-6-boronohexanoic acid (ABH) from Calbiochem (La Jolla, CA, USA). Antibodies against p-CaMKII (Cat. No. 12716), CaMKII (Cat. No. 3362), MLC20 (Cat. No. 3672), and p-MLC20 (Cat. No. 3674) were purchased from Cell Signaling Technology (Danvers, MA, USA). Arginase II (sc-20151), eNOS (sc-645), HSP60 (sc-1052), and α-actin (sc-56459) antibodies were purchased from Santa Cruz Biotechnology (Santa Cruz, CA, USA). p32 antiserum was obtained from Abcam, Co. (Cat. No. AB2991, Cambridge, MA). siRNA against arginase II (siArgII, sc‐29729) and scramble RNA (scmRNA, sc‐37007) were purchased from Santa Cruz Biotechnology. All reagents were purchased from Sigma (St. Louis, MO, USA) unless otherwise stated.

### Isolation of nLDL and cell culture

The nLDL (density 1.019–1.063 g ml^−1^) was prepared from the plasma of normocholesterolemic subjects by differential ultracentrifugation as previously described^7^. Isolated nLDL did not exhibit oxidative modification within 3 weeks compared with the oxidation of commercial nLDL and oxidized LDL from Intracel (Frederick, MD, USA), as determined by thiobarbiturate-reactive substance assays using malondialdehyde as a standard.

Human aortic smooth muscle cells (hAoSMCs) from four different batches were purchased from Lonza, Co. (Allendale, NJ, USA), maintained in medium 231 supplemented with SMGS (Cascade Biologics, Portland, OR, USA) according to the manufacturer’s protocol and treated with nLDL (final concentration, 100 µg/ml) after incubation in starvation medium (DMEM, 0.1% FBS, 100 U/ml penicillin, 100 µg/ml streptomycin) for 24 h. mAoSMCs of at least eight different batches were isolated from the thoracic and upper parts of the abdominal aorta of 10-week-old male mice (C57BL/6) as previously described^23^ with minor modifications. Briefly, the stripped aorta was prepared from the anesthetized mice and cut into 2-mm pieces, which were treated with type II collagenase (1 mg/ml, Invitrogen, Carlsbad, CA, USA) for 1 hr to remove the endothelial cells. The de-endothelialized aortic pieces were incubated with culture medium on gelatin (0.1%)-coated culture dishes for approximately 10 days. The cells were cultured in DMEM supplemented with 10% FBS, 100 U/ml penicillin, 100 µg/ml streptomycin, 8 mM HEPES, and 2 mM glutamine at 37 °C in a humidified 5% CO_2_ incubator. Before all experiments, isolated mAoSMCs at passage numbers 1–3 were maintained for 24 h in starvation medium to maintain their contractile phenotype. mAoSMCs were identified by their ‘spindle-shaped’ pattern and further confirmed by double staining using platelet endothelial cell adhesion molecule-1, a specific marker for endothelial cells, and α-smooth muscle actin, a specific marker for SMCs. All cells were stained with anti-α-smooth muscle actin antibodies. Each experiment was performed in at least two different batches of VSMCs depending on the availability of the cells.

### Animals

The study was approved in accordance with the Guide for the Care and Use of Laboratory Animals (Institutional Review Board, Kangwon National University). Studies were performed in accordance with the National Institutes of Health Guide for the Care and Use of Laboratory Animals. Three pairs of ArgII^−/−^ mice with a C57BL/6 background were gifted generously by Prof. Jaye Chin-Dusting to establish a colony. Mice were anaesthetized by 2.5% isoflurane in O_2_ and subjected to the following experiments. Aortic rings were studied from ten 10-week-old male C57BL/6 WT mice fed a normal diet (ND) and ten 10-week-old male ApoE^−/−^ mice (Daehan Biolink, Co.) fed a high-cholesterol diet (HCD, D12108C, Research Diet, Inc., USA) for 8 weeks^1,24^.

### Arginase activity

Arginase activity was determined from urea concentration measurements as described previously^4^.

### [Ca^2+^]m and [Ca^2+^]c measurement using confocal microscopy and flow cytometry

Direct assessment of the mitochondrial Ca^2+^ content ([Ca^2+^]m) was performed by an established loading procedure of the cells with calcein acetoxymethyl ester (calcein-AM, Thermo Fisher Scientific, Waltham, MA, USA) and CoCl_**2**_ for mitochondrial localization of calcein fluorescence^25^. Briefly, cells were loaded with 500 nM calcein-AM at 37 °C for 30 min in starved media. Calcein-AM generates free fluorescent intracellular calcein upon cleavage of the ester bond. CoCl_**2**_ was then added to the medium to quench the cytosolic calcein fluorescence, while intramitochondrial calcein allowed visualization of the mitochondria as bright fluorescent bodies. Subsequently, the cells were washed free of calcein-AM and CoCl_**2**_ and incubated in Tyrode’s modified solution (150 mM NaCl, 4 mM KCl, 2 mM CaCl_2_, 2 mM MgCl_2_, 10 mM HEPES, and 10 mM glucose). For detection of calcein fluorescence, a 470 nm excitation and a 510 nm emission filter were used. We also tested [Ca^2+^]m using Rhod-2 AM (Thermo Fisher Scientific, Co.). Briefly, cells were loaded with 2.5 μmol/L Rhod-2 AM at 37 °C for 1 h in starved media. Subsequently, the cells were washed free of Rhod-2 AM and incubated in Tyrode’s modified solution (NaCl 150 mmol/L, KCl 4 mmol/L, CaCl_2_ 2 mmol/L, MgCl_2_ 2 mmol/L, HEPES 10 mmol/L and glucose 10 mmol/L). For detection of Rhod-2 AM fluorescence, 552 nm excitation and 581 nm emission filters were used. MitoTracker green FM (Thermo Fisher Scientific, Co.) was incubated to confirm the mitochondria at 100 nmol/L for 1 h and imaged at 490 nm excitation and 516 nm emission. [Ca^2+^]c was monitored using Fluo-4 AM (100 nmol/L, 1 h, Thermo Fisher Scientific, Co.) at 494 nm excitation, and emission at 506 nm was detected. Intensity values were normalized according to the initial fluorescence values after subtraction of background using the Metamorph program (Molecular Probe).

[Ca^2+^]m and [Ca^2+^]c were also determined using flow cytometry (FACSCalibur). The fluorescence intensity for each sample was determined using CellQuest software. The Ca^2+^ level was determined by comparing the fold changes in the fluorescence intensities of treated cells versus control cells.

### siRNA treatment and knockdown of (p32)m

Serum-starved hAoSMCs were incubated in starvation medium containing sip32m (100 nM, 5′-TGT CTC CGT CGG TGT GCA GC-Cy5-3′), scm siRNA (100 nM, 5′-GCT GCA CAC CGA CGG AGA CA-Cy5-3′) or no oligonucleotide for 24 h without a transfection reagent. Dissected thoracic aorta from mice were incubated for 24 h in DMEM (2% FBS, penicillin (100 U/ml) and streptomycin (100 μg/ml)) containing sip32m (100 nM, 5′-TGT CTC CTT CCG TGT GCA GA-Cy5-3′) and scm siRNA (100 nM, 5′-CAG CAC AGC CCT GGA GCA CC-Cy5-3′).

### Mitochondria fractionation

Cells and de-endothelialized aortic segments were homogenized twice in subcellular fractionation buffer (250 mM sucrose, 20 mM HEPES, pH 7.4, 10 mM KCl, 1.5 mM MgCl_2_, 1 mM EDTA, 1 mM EGTA, and protease inhibitors) (Roche, Co., Basel, Switzerland) for 3 min and centrifuged at 1000 × *g* for 10 min to remove cell debris and unbroken cells. The supernatants were centrifuged at 21,000 × *g* for 45 min at 4 °C. The cytosolic (supernatant) and mitochondrial (precipitate) fractions containing 20 μg proteins were used for subsequent western blotting analyses of p32 protein expression.

### Western blot analysis

Cell extract and tissue extract were subjected to sodium dodecyl sulfate polyacrylamide gel electrophoresis (SDS-PAGE) and analyzed for the densitometry of bands using ImageJ from the National Institutes of Health (NIH)^4^.

### Fluorescence detection in aortic tissues

De-endothelialized aortic vessels treated with Cy5-conjugated sip32m and scm siRNA for 24 h were fixed in 4% formaldehyde, and frozen sections (5 μm) were used for the detection of Cy5 fluorescence. The slides were examined under a fluorescence microscope (Olympus) linked to a Clara EMCCD digital camera (Andor Technol.) and collected using MetaMorph software.

### Polyamine analysis

Intracellular concentrations of L-arginine and polyamine, spermine, spermidine, and putrescine were determined by HPLC using pre-column derivatization with o-phthalaldehyde (OPA) according to a modification of previously published methods^26^. Briefly, L-arginine (100 μM) and polyamine (30 μM/each) were added to cell lysate (0.1 mM) as an internal standard. The samples were extracted on solid-phase extraction cartridges (CBA Bond elute, Varian, Yverdon, Switzerland), and the recovery rate was 87.5 ± 3.9% for L-arginine. Eluates were dried over nitrogen and resuspended in double-distilled water for HPLC analysis. HPLC was performed on a computer-controlled Waters chromatography system (M600E) consisting of an automatic injector (M7725i, Waters Co., Easton, MA, USA) and a fluorescence detector (FP-1520, Jasco Co., Easton, MA, USA). Samples were incubated for 1 min with OPA reagent (5.4 mg/ml OPA in borate buffer, pH 8.4, containing 0.4% 2-mercaptoethanol) before automatic injection to the HPLC. The OPA derivative of L-arginine was separated on a 150 × 4.6 mm-5 μm Zorbax Eclipse (Agilent Technologies, Santa Clara, CA, USA) XDB-C18 column with the fluorescence detector set at Ex 340 nm and Em 450 nm. Samples were eluted from the column with 0.96% citric acid/methanol (70:30), pH 6.8 at a flow rate of 1.5 ml/min.

### Vessel reactivity assay

The thoracic aorta was dissected from anesthetized mice (C57BL/6) with isoflurane, and fat and connective tissues were cleaned. The aorta was cut into 1.5-mm rings, and the endothelium was removed gently using a wooden stick. Then, the samples were suspended between two wire stirrups (150 μm) in a myograph (Multi myograph system DMT-620, Aarhus, Denmark) in 10 ml Krebs-ringer solution (95% O_2_, 5% CO_2_, pH 7.4, 37 °C). One stirrup was connected to a three-dimensional micromanipulator and the other to a force transducer. The rings were passively stretched at 10-min intervals in 100 mg increments to reach optimal tone (600 mg). After the arterial rings had been stretched to their optimal resting tone, the contractile response to 100 mM KCl was determined. The response to a maximal dose of KCl was used to normalize the agonist responses across vessel rings. Dose responses to the vasoconstrictor PE (10^−10^–10^−6^ M) and NE (10^−9^–10^−5^ M) were performed.

### Statistical analyses

All data are reported as the mean ± SEM. Statistical significance was determined by the Bonferroni-corrected unpaired *t-*test for unpaired values. A *p* value of <0.05 was used as the criterion for statistical significance. Dose responses were analyzed by 2-way analysis of variance (ANOVA) using GraphPad Prism 4.0 Software.

## Results

### ArgII activity regulates [Ca^2+^]m and [Ca^2+^]c in nLDL-stimulated hAoSMCs

**Fig. 1:**
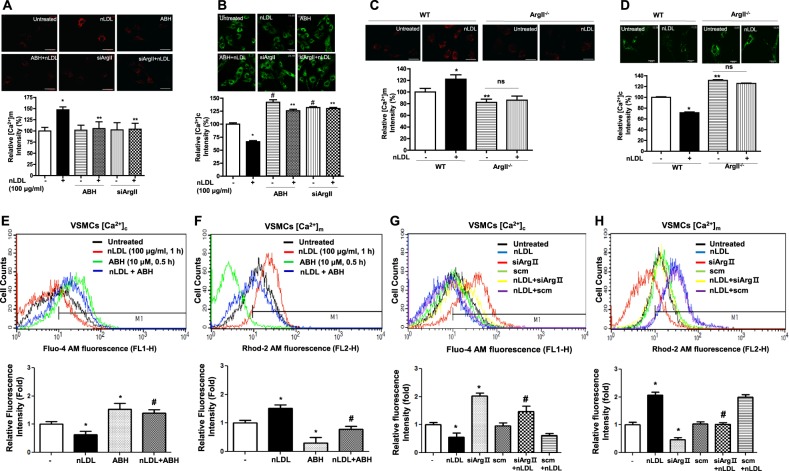
Arginase inhibition attenuates [Ca^2+^]m uptake and elevates [Ca^2+^]c levels in nLDL-stimulated hAoSMCs. **a** [Ca^2+^]m was measured with nLDL treatment for 30 min in the presence of an arginase inhibitor, ABH (10 μM), and siRNA incubation against ArgII (SiArgII, 100 nM, 24 h) using the Rhod-2 AM method. * vs. untreated, *P* < 0.01. ** vs. nLDL, *P* < 0.01. **b** With the same treatments, [Ca^2+^]c levels were analyzed by staining cells with Fluo-4 AM. * vs. untreated, *P* < 0.01. # vs. untreated, *P* < 0.01. ** vs. nLDL, *P* < 0.01. mAoSMCs were isolated from the aortas of WT and ArgII^−/−^ mice, and [Ca^2+^]m (C) and [Ca^2+^]c **d** levels were examined with or without nLDL treatment. * vs. WT + untreated, *P* < 0.01, ** vs. WT + untreated, *P* < 0.01. All statistical analyses were performed using an unpaired *t*-test. *n* = 3 independent experiments, and fluorescent intensity was quantified from 9–15 images per experiment. Bars indicate 10 μm (**a**, **b**, **c**) and 50 μm (**d**). FACS analyses were performed to confirm the data from microscopic analyses (E-H). Arginase inhibition with ABH (**e**, **f**) and siArgII (**g**, **h**) increased [Ca^2+^]c and reciprocally decreased [Ca^2+^]m. * vs. untreated, *P* < 0.01. # vs. nLDL, *P* *<* 0.01. *n* = 3 independent experiments

We first wished to determine the effect of ArgII inhibition on mitochondrial Ca^2+^ uptake in cultured hAoSMCs (Supplementary Fig. 1A). nLDL stimulation for 30 min resulted in increased [Ca^2+^]m (Fig. 1a, * vs. untreated, 147 ± 6.6 vs. 100 ± 7.9%, *P* < 0.01), which was abolished by pretreatment with the ArgII inhibitor ABH and small interfering RNA against ArgII (siArgII) (Fig. 1a, ** vs. nLDL only, *P* < 0.01. Supplementary Fig. 1A). Conversely, incubation of nLDL reduced [Ca^2+^]c, and inhibition of ArgII with ABH and siArgII significantly increased [Ca^2+^]c in the nLDL-treated conditions (Fig. 1b, * vs. untreated, 66.2 ± 6.7 vs. 100.0 ± 8.3%, *P* < 0.01, # vs. untreated, 142.0 ± 15.2 (ABH) and 132.0 ± 5.4 (siArgII) vs. 100.0 ± 8.3%, *P* < 0.01, ** vs. nLDL, 125.8 ± 9.5 (ABH) and 130.0 ± 6.8 (siArgII) vs. 66.2 ± 6.7%, *P* < 0.01). To further confirm the effect of Arg inhibition on [Ca^2+^]m, we isolated aortic smooth muscle cells (mAoSMCs) from WT and ArgII^−/−^ mice (Supplementary Fig. 1B). Stimulation of isolated mAoSMCs from WT mice with nLDL increased [Ca^2+^]m (Fig. 1c, * vs. untreated, 138.6 ± 11.6 vs. 100 ± 11.2%, *P* < 0.01). The [Ca^2+^]m level in mAoSMCs from ArgII^−/−^ mice was significantly lower (Fig. 1c, ** vs. WT + untreated, 82.2 ± 5.5 vs. 100 ± 11.2%, *P* < 0.05) and was not changed by nLDL stimulation (Fig. 1c, ArgII^−/−^ vs. ArgII^−/−^+nLDL, 82.2 ± 5.5 vs. 86.2 ± 7.1%, ns). However, [Ca^2+^]c in mAoSMCs from WT mice was decreased by nLDL treatment (Fig. 1d, * vs. untreated, 71.5 ± 8.1 vs. 100 ± 6.5%, *P* < 0.01), and [Ca^2+^]c in mAoSMCs from ArgII^−/−^ mice was higher (** vs. WT + untreated, 100 ± 6.5 vs. 131.4 ± 6.1%, *P* < 0.01) and not affected by nLDL (ArgII^−/−^ vs. ArgII^−/−^+nLDL, ns). The results from microscopic images were confirmed again with FACS analysis (Fig. 1e–h, * vs. untreated, *P* < 0.01; # vs. nLDL only, *P* < 0.01).

### Downregulation of ArgII activity induces CaMKII/MLC20 phosphorylation, which contributes to vessel constriction in de-endothelialized aortas

**Fig. 2:**
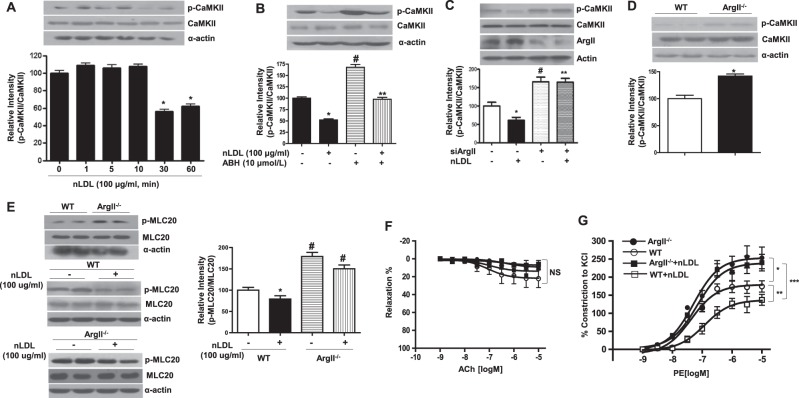
Arginase II activity regulates CaMKII/MLC20 phosphorylation and vessel constriction. Western blotting analysis was performed to determine CaMKII phosphorylation after the treatment of nLDL (100 μg/ml) for different time points (**a**, * vs. untreated, *P* < 0.01), after the stimulation of nLDL in the pretreatment (30 min) of ABH (**b**) and siArgII (**c**, * vs. untreated, *P* < 0.01; # vs. untreated, *P* < 0.01; ** vs. nLDL, *P* < 0.01), and in the isolated mAoSMCs from WT and ArgII^−/−^ mice (**d**, * vs. WT, *P* < 0.01). MLC-20 phosphorylation was also examined after nLDL stimulation using isolated mAoSMCs (**e**, * vs. WT+untreated, *P* < 0.05; # vs. WT+untreated or nLDL treated, *P* < 0.01). Aortic rings were de-endothelialized and had no response to Ach (**f**, NS not significant). Vessel reactivity of de-endothelialized rings against a vasoconstrictive agonist, PE (10^–9^–10^–5^ M), was tested (**g**, *, WT vs. ArgII^−/−^, *P* < 0.01; **, WT vs. WT + nLDL, *P* < 0.01; ***, WT + nLDL vs. ArgII^−/−^ + nLDL, *P* < 0.01). *n* = 4 independent experiments

To assess the hypothesis that nLDL treatment decreased [Ca^2+^]c, we measured the activation of a Ca^2+^-dependent protein kinase, CaMKII, which was related to VSMC constriction. As shown in Fig. 2a, nLDL stimulation attenuated CaMKII phosphorylation in a time-dependent manner. Next, we tested whether downregulation of ArgII activity recovered nLDL-dependent CaMKII dephosphorylation. ArgII downregulation with ABH (Fig. 2b, untreated, 100 ± 5.1, nLDL, 52 ± 4.2, ABH, 168 ± 11.3, nLDL+ABH, 97.6 ± 7.1 Arbitrary unit (AU)) and siArgII (Fig. 2c, untreated, 100 ± 10.2, nLDL, 60.9 ± 8.5, siArgII, 166.1 ± 12.6, nLDL+siArgII, 165.2 ± 10.1 AU) induced an increase in CaMKII phosphorylation, and CaMKII phosphorylation was enhanced in de-endothelialized aortas from ArgII^−/−^ (Fig. 2d, WT, 100 ± 11.2, ArgII^−/−^, 179 ± 16.8 AU). Elevated CaMKII phosphorylation by ArgII downregulation was associated with MLC20 phosphorylation. As shown in Fig. 2e, nLDL incubation significantly reduced MLC20 phosphorylation in de-endothelialized aortas from WT, but MLC20 phosphorylation was enhanced in de-endothelialized aortas from ArgII^−/−^ and maintained at high levels even with nLDL stimulation (WT, 100 ± 11.2, WT + nLDL, 79.1 ± 13.2, ArgII^−/−^, 179.0 ± 16.8, ArgII^−/−^ ± nLDL, 150.1 ± 15.3 AU). MLC20 phosphorylation in de-endothelialized vessels (Fig. 2f) was clearly correlated with phenylephrine (PE)-dependent vasoconstriction activity (Fig. 2g, Emax, WT, 174.1 ± 31.2, ArgII^−/−^, 253.3 ± 42.4, WT+nLDL, 137.0 ± 21.4, ArgII^−/−^+nLDL, 240.1 ± 32.4%).

### Knockdown of mitochondrial p32 reduces nLDL-induced Ca^2+^ uptake into mitochondria, resulting in CaMKII phosphorylation

**Fig. 3:**
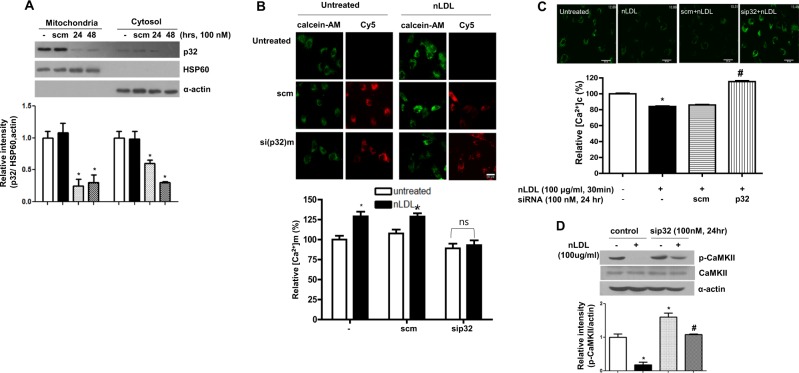
Mitochondrial p32 plays a key role in mitochondrial Ca^2+^ uptake in nLDL-stimulated hAoSMCs. sip32 was incubated with serum-starved hAoSMCs and reduced [p32]m after 24 h of incubation (**a**, * vs. untreated control, *P* < 0.01). HSP60 is a marker protein for the mitochondrion, and actin is for cytosol. Cells were incubated with Cy5-tagged sip32 or scramble (scm) siRNA for 24 h, and [Ca^2+^]m was imaged (**b**, upper) and quantified (**b**, lower, *vs. untreated, *P* < 0.05). Translocation of siRNA into cells was confirmed by imaging at the Cy5 wavelength. Representative image from three independent experiments. sip32 treatment restored reduced [Ca^2+^]c (**c**) and decreased CaMKII phosphorylation by nLDL treatment (**d**). * vs. untreated control, *P* < 0.01, # vs. nLDL, *P* < 0.01. All experiments are representative of 3–4 independent experiments

To determine a Ca^2+−^mobilizing protein that regulates nLDL-dependent Ca^2+^ uptake into mitochondria, scramble (scm) and siRNA oligonucleotides against mitochondrial p32 (sip32m) mRNA were designed. Incubation of sip32m with hAoSMCs at 100 nM for 24 h reduced the p32m level without affecting the p32c level (Fig. 3a). Both sip32m and scm siRNA were taken up into cells (Fig. 3b upper, from Cy5 image), and sip32m abolished Ca^2+^ transition to the mitochondria by nLDL stimulation (Fig. 3b, lower, * vs. untreated, *P* < 0.01; ns, not significant). Furthermore, as revealed by the sip32m-dependent increase in [Ca^2+^]c (Fig. 3c), sip32m restored the attenuated CaMKII phosphorylation by nLDL stimulation (Fig. 3d). However, the mitochondrial permeability transition pore and mitochondrial Ca^2+^ uniporter were not involved in the mitochondrial Ca^2+^ transition in nLDL-stimulated hAoSMCs (Supplementary Fig. 2).

### Spermine induces a p32-dependent increase in [Ca^2+^]m that attenuates CaMKII phosphorylation

**Fig. 4:**
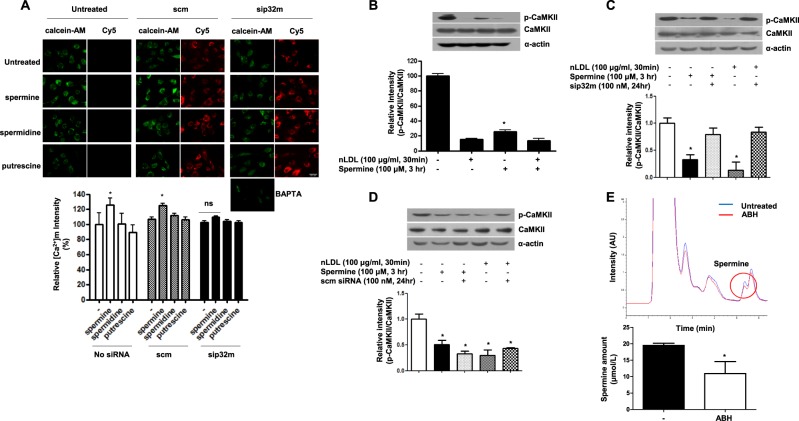
Spermine is a primary activator of mitochondrial Ca^2+^ uptake through mitochondrial p32. hAoSMCs were treated with Cy5-conjugated sip32 and scm siRNA for 24 h and stimulated with polyamine, putrescine, spermidine, and spermine (each 100 μM, 3 h). [Ca^2+^]m levels were imaged by CoCl_2_-quenched calcein-AM fluorescence, and the translocation of siRNA into cells was confirmed by Cy5 imaging (**a**, upper). Spermine treatment induced an increase in [Ca^2+^]m that prevented incubation of sip32 (**a**, lower, * vs. untreated, *P* < 0.05). *n* = 3 independent experiments and 10–12 different images were obtained in each experiment. **b** Spermine decreased CaMKII phosphorylation in nLDL-treated hAoSMCs (* vs. untreated, *P* < 0.05). Incubation of sip32 restored nLDL- and spermine-induced decreases in CaMKII phosphorylation (**c**), but scm siRNA had no effect on CaMKII phosphorylation (**d**). All blots are representative of three independent experiments. **e** Arginase inhibition with ABH (10 μM) reduced intracellular spermine concentration (top, representative HPLC chromatogram, * vs. untreated, *P* < 0.05). *n* = 4 experiments

To test whether ArgII activity impacts p32-dependent mitochondrial Ca^2+^ uptake, cells were incubated with polyamines, spermine, spermidine, and putrescine, without siRNA. Interestingly, only spermine treatment induced an increase in [Ca^2+^]m (Fig. 4a, * vs. untreated, 126 ± 8.9 vs. 100 ± 12.5%, *P* < 0.05), while spermidine and putrescine had no effect. To confirm the association between spermine-dependent mitochondrial Ca^2+^ uptake and p32m, cells were preincubated with sip32m and scm siRNA and then stimulated with polyamine. Indeed, sip32m treatment prevented spermine-induced mitochondrial Ca^2+^ uptake (Fig. 4a, 104 ± 5.9 vs. 102 ± 4.6%, ns), but scm treatment produced no effect (Fig. 4a, middle column, * vs. untreated, 106 ± 4.7 vs. 124 ± 5.8%, *P* < 0.01). We next tested whether the incubation of spermine affects CaMKII phosphorylation. As shown in Fig. 4b, spermine treatment significantly reduced CaMKII phosphorylation (* vs. untreated, 25 ± 3.9 vs. 100 ± 5.1 AU, *P* < 0.01). The effect of spermine on CaMKII phosphorylation was prevented by sip32m incubation (Fig. 4c), but scm did not affect CaMKII activation (Fig. 4d). Because spermine-induced mitochondrial Ca^2+^ transition through p32m and arginase inhibition decreased mitochondrial Ca^2+^ levels, we tested whether arginase inhibition had an effect on spermine levels. As shown in Fig. 4e, arginase inhibition reduced intracellular spermine levels (* vs. untreated, 19.4 ± 0.6 vs. 10.9 ± 3.6 μmol/L, *P* < 0.01). Therefore, we suggest here that ArgII inhibition attenuates [Ca^2+^]m by decreasing the level of spermine inducing the p32-dependent transition of cytosolic Ca^2+^ into the mitochondria.

### sip32m incubation enhances responses to vasoconstrictors

**Fig. 5:**
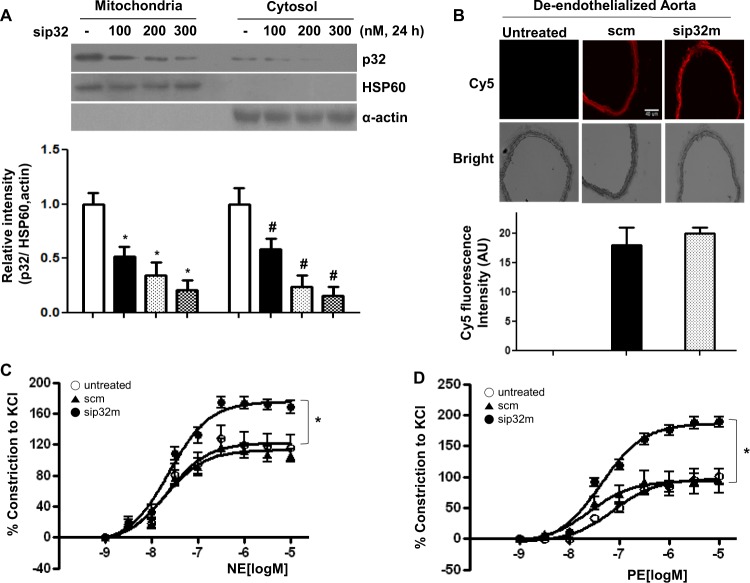
Downregulation of p32 enhances vessel constriction in de-endothelialized aortic vessels from WT mice. sip32 (100 and 300 nM) and scm siRNA (100 nM) were incubated with de-endothelialized aortas from WT mice for 24 h. sip32 showed a decrease in protein levels (**a**), and siRNAs were moved into VSMCs as shown in the Cy5 image. The intensity of fluorescence is shown in a bar graph (**b**). Vascular tension assays with vasoconstrictive agonists, NE (**c**) and PE (**d**), were performed with siRNA-treated de-endothelialized aortic vessels. Incubation of sip32 enhanced vessel constriction to both agonists. *, *P* < 0.01, *n* = 4 mice

We next tested whether sip32m could regulate VSMC constriction. First, sip32m incubation with de-endothelialized rings from WT mice significantly reduced the p32m level at 100 nM, but scm siRNA had no effect on the protein level (Fig. 5a, * vs. untreated, *P* < 0.01; # vs. untreated, *P* < 0.01). To examine the movement of Cy5-conjugated siRNA into cells, the vessels were imaged with Cy5 fluorescence (Fig. 5b). We observed its transfer into VSMCs. In the vascular tension assay against agonists, norepinephrine (NE) and phenylephrine (PE), sip32m incubation enhanced the Emax values to NE (Fig. 5c, *, untreated vs. sip32m, 122.4 ± 6.9 vs. 175.6 ± 4.3%, *P* < 0.01) and PE (Fig. 5d, *, untreated vs. sip32m, 97.3 ± 4.5 vs. 186.8 ± 4.1%, *P* < 0.01). scm siRNA incubation resulted in no difference compared to the untreated rings.

### Increased Arg activity is associated with changes in [Ca^2+^]m, [Ca^2+^]c, and p32m levels in ApoE^−/−^ mice fed an HCD

**Fig. 6:**
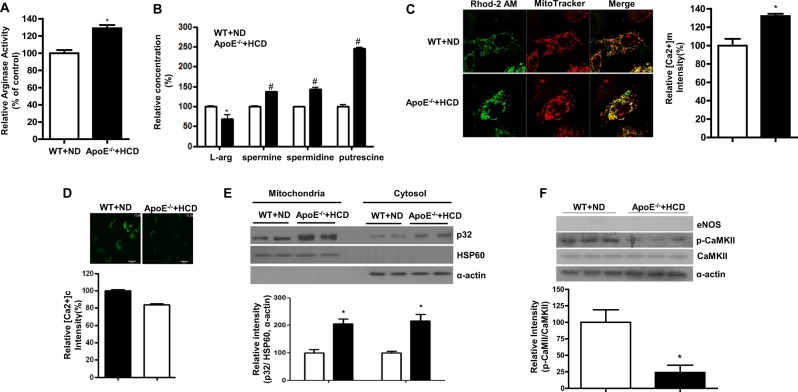
Intracellular spermine and mitochondrial p32 levels were upregulated in HCD-fed ApoE^−/−^ mice. Arginase activity was increased in de-endothelialized aortas from ApoE^−/−^ mice (**a**, *, *P* < 0.01), the intracellular concentration of L-arg was decreased and the spermine level was increased (**b**, * vs. WT+ND, *P* < 0.05; # vs. WT+ND, *P* < 0.01, *n* = 3 mice). mAoSMCs were isolated from aortas from WT mice fed an ND and ApoE^−/−^ mice fed an HCD for 8 weeks. [Ca^2+^]m and [Ca^2+^]c levels were measured by staining cells with Rhod-2 AM and Fura-2 AM, respectively. [Ca^2+^]m was increased (**c**, *, *P* < 0.05), but [Ca^2+^]c was decreased (**d**, *, *P* < 0.05) in mAoSMCs from ApoE^−/−^ mice fed an HCD. Mitochondria fractionation with de-endothelialized aortas showed an increase in the [p32]m level (E, * vs. WT+ND, *P* < 0.01) and a decrease in CaMKII phosphorylation (F, * vs. WT+ND, *P* < 0.01). *n* = 3 independent experiments

Compared to the results from WT mice, we examined whether the expression of p32m was altered in an atherogenic mouse model (ApoE^−/−^) fed a high-cholesterol diet (HCD). Arg activity was increased in de-endothelialized aortas of ApoE^−/−^ mice fed an HCD (Fig. 6a, * vs. WT+ND, 120 ± 1 vs. 100 ± 2%, *P* < 0.01). In addition, the level of L-arginine was decreased (* vs. WT + ND, 68.7 ± 10 vs. 100 ± 1%, *P* < 0.01), while the concentrations of polyamine, spermine (# vs. WT + ND, 138.6 ± 0.9 vs. 100 ± 3%, *P* < 0.01), spermidine (# vs. WT+ND, 148.9 ± 5% vs. 100 ± 2%, *P* < 0.01), and putrescine (# vs. WT+ND, 242.7 ± 3% vs. 100 ± 4%, *P* < 0.01) were increased (Fig. 6b). Furthermore, [Ca^2+^]m was also elevated (Fig. 6c, * vs. WT + ND, 111.8 ± 2 vs. 100 ± 2%, *P* < 0.05) and [Ca^2+^]c was reduced (Fig. 6d, * vs. WT + ND, 84.0 ± 4.7 vs. 100 ± 5.5%, *P* < 0.05) in isolated mAoSMCs from ApoE^−/−^ fed an HCD, which was consistent with the augmented p32m expression (Fig. 6e, * vs. WT + ND, 321 ± 26.1 vs. 100 ± 5.8 AU, *P* < 0.01) and attenuated CaMKII activation (Fig. 6f, * vs. WT + ND, 24 ± 11 vs. 100 ± 19 AU, *P* < 0.01) in de-endothelialized aortas of ApoE^−/−^ mice fed an HCD. The increased p32m expression in ApoE^−/−^ fed an HCD might be dependent on the diet type because mice fed an ND did not show changes in p32m expression (Supplementary Fig. 3).

### Effect of p32m on vasoconstriction in ApoE^−/−^ mice fed an HCD

**Fig. 7:**
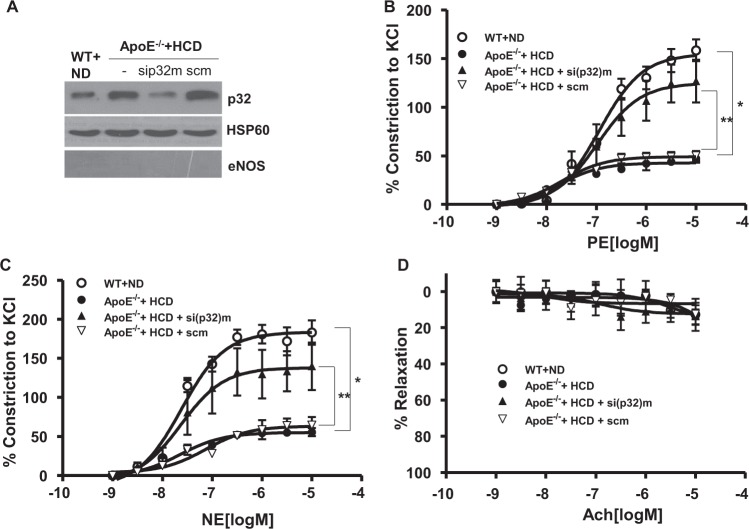
sip32 treatment restores vasoconstriction activity in de-endothelialized vessels from HCD-fed ApoE^−/−^ mice. De-endothelialized aortas from WT mice fed an ND and ApoE^−/−^ mice fed an HCD for 8 weeks were incubated with sip32 and scm siRNA. Incubation of sip32 resulted in a decrease in mitochondrial protein levels (**a**, representative blot from three independent experiments). In a vascular tension assay using de-endothelialized aortas with vasoconstrictive agonists, PE (**b**) and NE (**c**), the decreased constriction in aortas from ApoE^−/−^ mice fed an HCD was restored with incubation of sip32 (*, *P* < 0.01, **, *P* < 0.01). *n* = 4 mice. All groups showed no responses to Ach (**d**)

In de-endothelialized aortas from WT fed an ND and ApoE^−/−^ mice fed an HCD, sip32m treatment significantly reduced the p32m level (Fig. 7a). With the same treatment, accumulated vasoconstrictor responses to PE and NE were constructed. The attenuated vasoconstriction responses to PE in aortas from ApoE^−/−^ mice fed an HCD compared to WT mice fed an ND were improved with sip32m incubation (Fig. 7b, *, WT + ND vs. ApoE^−/−^ + HCD, 158.5 ± 22.3 vs. 47.1 ± 8.1%, *P* < 0.01, **, ApoE^−/−^ + HCD vs. ApoE^−/−^ + HCD+sip32 m, 47.1 ± 8.1 vs. 126.9 ± 43.9%, *P* < 0.01). Consistent with these data, the responses to NE were similar to PE responses (Fig. 7c, *, WT + ND vs. ApoE^−/−^ + HCD, 182.9 ± 31.2 vs. 55.5 ± 10.2%, *P* < 0.01, **, ApoE^−/−^ + HCD vs. ApoE^−/−^ + HCD + sip32 m, 55.5 ± 10.2 vs. 139.7 ± 60.7%, *P* < 0.01). All groups showed no response to an endothelium-dependent vasorelaxant, acetylcholine (Ach, Fig. 7d).

## Discussion

In this study, we demonstrated for the first time that the activity of ArgII, a mitochondria-targeting Arg isoform, regulates mitochondrial Ca^2+^ transition and CaMKII activation via spermine/p32m-dependent mitochondrial Ca^2+^ uptake in VSMCs. Furthermore, downregulation of p32m using siRNA resulted in increased [Ca^2+^]c and CaMKII/MLC20 phosphorylation and enhanced vasoconstriction in de-endothelialized aortic vessels. In de-endothelialized aortas from ApoE^−/−^ mice fed an HCD, both Arg activity and spermine levels increased. Interestingly, p32m was upregulated and [Ca^2+^]m was augmented in mAoSMCs from atherogenic mice. Consistent with the above data, CaMKII phosphorylation was decreased and vascular tension in response to vasoconstrictors was attenuated in de-endothelialized aortas from ApoE^−/−^ mice fed an HCD. However, incubation with sip32m restored responses to the vasoconstrictors.

Increased Arg activity contributes to the pathophysiology of various disease processes. Vascular endothelial Arg leads to endothelial dysfunction by substrate depletion and contributes to vascular diseases^24,27–30^. Consistent with these observations, long-term administration of L-arginine in some preclinical studies has been shown to prevent atherosclerosis and myointimal hyperplasia and enhance angiogenesis^31–33^. However, in patients with peripheral arterial disease, long-term L-arginine administration resulted in increased levels of ornithine and urea and did not improve vascular reactivity^34^. This discrepancy may be partly explained by our study demonstrating that increased Arg activity provoked the production of spermine, which may induce mitochondrial Ca^2+^ uptake via p32m. This phenomenon is a reason for mitochondrial dysfunction. Increased Ca^2+^ uptake into mitochondria becomes a pathological stimulus by promoting ROS generation, cytochrome C release, and apoptosis ^35–37^.

Mitochondrial Ca^2+^ transport plays a central role in cellular physiology and pathophysiology. Ca^2+^ influx from the cytosol can control the rate of mitochondrial energy production through activation of pyruvate dehydrogenase, isocitrate dehydrogenase, and α–ketoglutarate dehydrogenase in the Krebs cycle^38^ and through regulation of FoF1ATPase^39^ and adenine nucleotide translocase^40^ activity in the electron transport chain. Thus, increased Ca^2+^ uptake into the mitochondria in nLDL-stimulated VSMCs appears to be a natural phenomenon, as nLDL primarily serves to supply lipids and is then catabolized through β-oxidation in mitochondria to acetyl-CoA, which is utilized for the production of energy (ATP). Our study showed that nLDL stimulation exerted an increase in [Ca^2+^]m that was prevented by Arg inhibition (Fig. 1a) and ArgII gene deletion (Fig. 1c). However, [Ca^2+^]c was decreased by nLDL stimulation that was also recovered by downregulation of Arg activity (Fig. 1b, d) and resulted in activation of a Ca^2+^-sensitive protein kinase, CaMKII (Fig. 2). In this study, we illustrate a mechanism in which mitochondrial dysfunction is an early phenomenon of cardiovascular diseases initiated by hypercholesterolemia. Our findings are consistent with previous demonstrations that the mitochondria decode and shape cellular Ca^2+^ signals^40–42^ by uptake and release of Ca^2+^ ions to modulate cell metabolism and cell death^43^. The main transporters contributing to rapid Ca^2+^ uptake into the mitochondria are reported^44^; herein, we present for the first time a role for p32m as a novel mitochondrial Ca^2+^ transporter. Indeed, p32m could form a doughnut-shaped trimer with 48 amino acids, aspartic and glutamic acids, distributed on the surface of the trimer. With its acidic surface, p32m has been suggested as a high-capacity divalent cation storage protein modulating mitochondrial cation concentrations^45^. Consistent with this assumption, we confirmed that p32m is involved in Ca^2+^ uptake using siRNA (Fig. 3b). Furthermore, p32m-dependent entry of Ca^2+^ into the mitochondria was enhanced with spermine treatment (Fig. 4a), and spermine pretreatment attenuated CaMKII phosphorylation (Fig. 4b). These observations were consistent with a previous report that spermine can play an important role in the regulation of the free cytoplasmic Ca^2+^ concentration and of the free Ca^2+^ concentration in the mitochondrial matrix, while the responsible channel protein was not identified^46^. Increased [Ca^2+^]m was shown to regulate cell metabolism^38^, and p32m was shown to be a regulator for maintenance of oxidative phosphorylation^21^. In tumor cells, p32m knockdown cells shift their metabolism from oxidative phosphorylation toward glycolysis^21^. Based on these results, we wanted to confirm the function of p32m in Ca^2+^-dependent contraction in VSMCs. As shown in Fig. 5, sip32m treatment enhanced the vasoconstriction responses to agonists through [Ca^2+^]c intensification. In VSMCs from ApoE-null mice fed an HCD, we found that the p32m levels were upregulated and [Ca^2+^]m was augmented (Fig. 6c and E). Indeed, Arg activity increased in ApoE^−/−^ mice fed an HCD, spermine concentration was high, and CaMKII phosphorylation was significantly attenuated compared to WT mice fed an ND (Fig. 6a, b, and F). Furthermore, the downregulation of increased p32m using sip32m restored attenuated contraction responses (Fig. 7). These results suggested that p32m expression may be regulated by Arg activity because p32m was downregulated in ArgII^−/−^ and upregulated in ApoE^−/−^.

In VSMCs, intracellular Ca^2+^ concentration to maintain basal vascular tone is modulated by Ca^2+^ release from the intracellular stores in the sarcoplasmic reticulum, and Ca^2+^ entry from the extracellular space through plasma membrane Ca^2+^ channels. Here, we present a novel mechanism underlying the regulation of [Ca^2+^]c by inhibiting Ca^2+^ uptake into mitochondria in nLDL-stimulated VSMCs. Increased [Ca^2+^]c activates specific protein kinases and phosphatases involved in VSMC contraction and relaxation. Indeed, Ca^2+^/calmodulin-dependent kinase II (CaMKII) is activated by elevated [Ca^2+^]c and autophosphorylated to participate in the cell cycle and growth regulation in VSMCs. In addition, CaMKII has been implicated as a regulator of VSMC contraction. Consistent with these observations, ArgII^−/−^ and p32m knockdown demonstrated augmented activation of CaMKII and enhanced vasoconstrictive responses to agonists in de-endothelialized vessels.

In this current study, two major points should be explained. The first is why [Ca^2+^]c was maintained at higher levels by downregulating Arg activity than that of untreated cells (Fig. 1b), although Arg inhibition reduced the increases in [Ca^2+^]m in nLDL-stimulated hAoSMCs to untreated levels. A possible reason is L-arginine content. Temporary inhibition of Arg activity definitely elicited a decrease in intracellular spermine amount and an increase in intracellular L-arginine levels^47^. Interestingly, incubation of cells with L-arginine increased intracellular [Ca^2+^]c levels (data not shown). Therefore, higher [Ca^2+^]c by transient inhibition of Arg activity (Fig. 1b) may be attributed to increased intracellular L-arginine contents. The second explanation is that [Ca^2+^]m in mAoSMCs from ArgII^−/−^ mice was maintained at lower levels than that of WT cells and not increased under nLDL-stimulated conditions. As shown in Supplementary Fig. 4, p32m levels were clearly downregulated, and PE-dose responses were attenuated in de-endothelialized aortas of ArgII^−/−^ mice. Therefore, the nLDL-dependent Ca^2+^ transition through p32m may be attenuated and then could induce a reciprocal increase in [Ca^2+^]c.

In conclusion, we demonstrated that ArgII activity, coupled with spermine production, contributes to mitochondrial dysfunction through increased p32m-dependent mitochondrial Ca^2+^ uptake. In particular, p32m levels were upregulated, and [Ca^2+^]m levels were increased in VSMCs from ApoE^−/−^ mice. Finally, downregulation of p32m using the siRNA technique augmented CaMKII phosphorylation and agonist-induced vasoconstriction activity.

## Supplementary information

Supplemental figures
