# Supplementary material for: Arginase II activity regulates cytosolic Ca2+ level in a p32-dependent manner that contributes to Ca2+-dependent vasoconstriction in native low-density lipoprotein-stimulated vascular smooth muscle cells
Source: Exp Mol Med. 2019 Jun 3;51(6):60. doi: 10.1038/s12276-019-0262-y (PMC6545325; doi:10.1038/s12276-019-0262-y)
Supplement: Supplementary file 1 — Supplemental figures [file 12276_2019_262_MOESM1_ESM.pdf]

Suppl. Fig. 1.

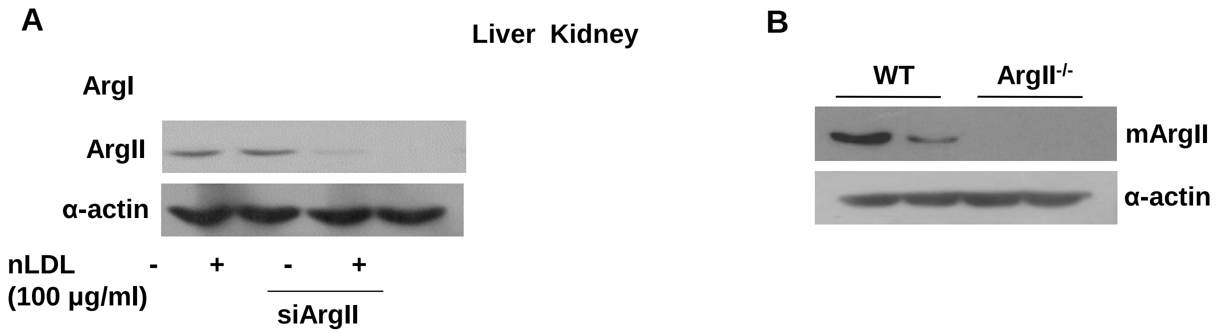

Supplemental Fig. 1. (A) ArgII isoform was dominantly expressed in hAoSMCs and specific siRNA against human ArgII downregulated ArgII protein expression. Liver and kidney lysates were used controls for ArgI and ArgII protein. (B) ArgII expression was confirmed in ArgII$^{-/-}$ mice.

Suppl. Fig. 2.

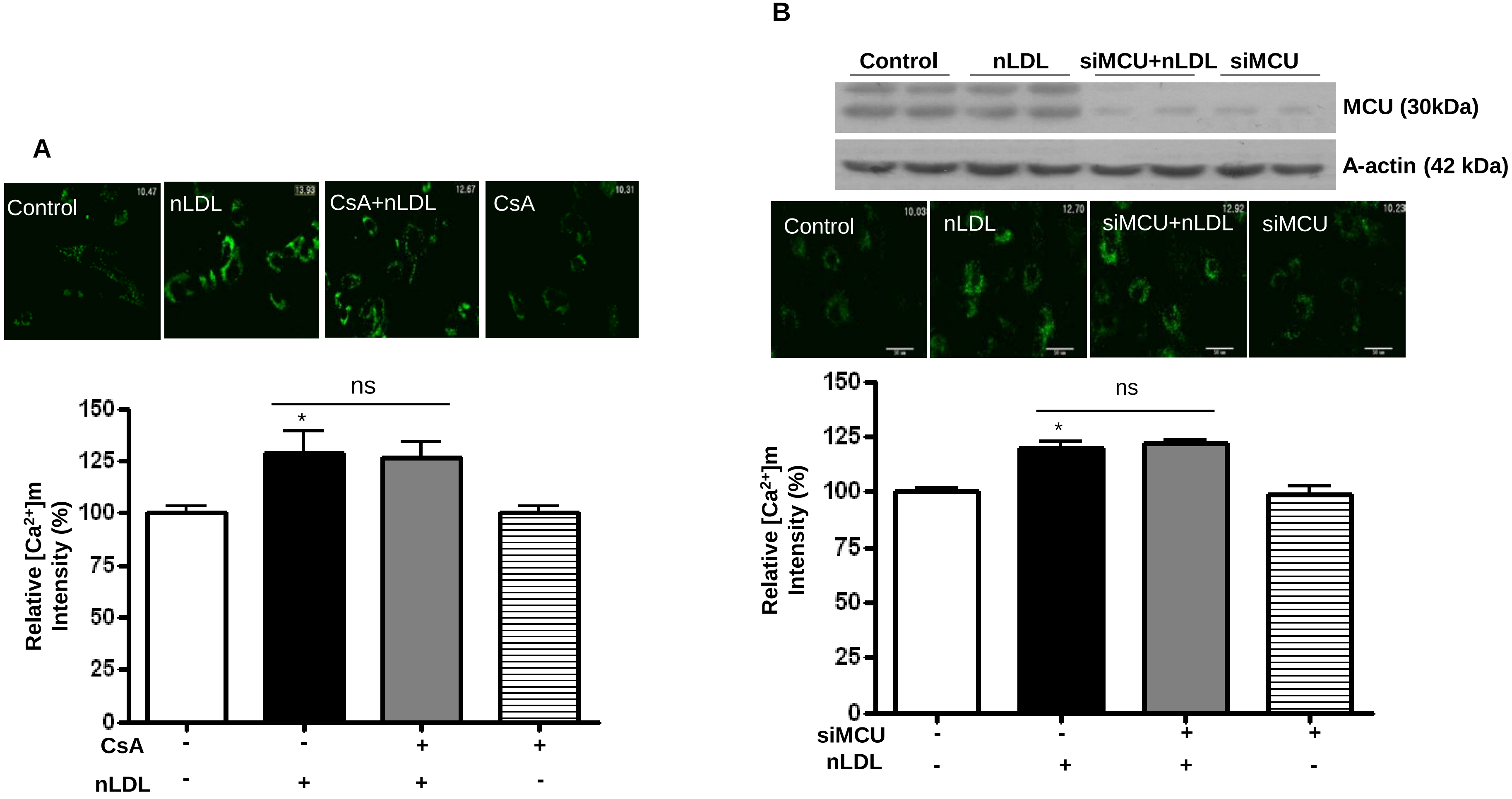

Supplemental Fig. 2. Effect of mitochondrial Ca^{2+} channels on nLDL-induced Ca^{2+} transition to mitochondria.

A. CsA(cyclosporine A) as an inhibitor to mitochondrial permeability transition pore was pretreated (10 μM, 1 hrs) and $[Ca^{2+}]$m level was measured after nLDL stimulation. CsA had no effect on nLDL-induced Ca^{2+} transition to mitochondria (* vs. untreated, P<0.01, ns, not significant).

B. Incubation of siRNA (5'-UCG ACC UAG AGA AAU ACA AU-3', 100 nM, 24 hrs) against mitochondrial Ca^{2+} uniporter (MCU) showed downregulation of MCU protein levels (B, Upper). siMCU incubation (100 nM, 24 hrs) had no effect on nLDL-dependent Ca^{2+} transition to mitochondria (B, lower). n=3 independent experiments and 10 images per each experiment.

Suppl. Fig. 3.

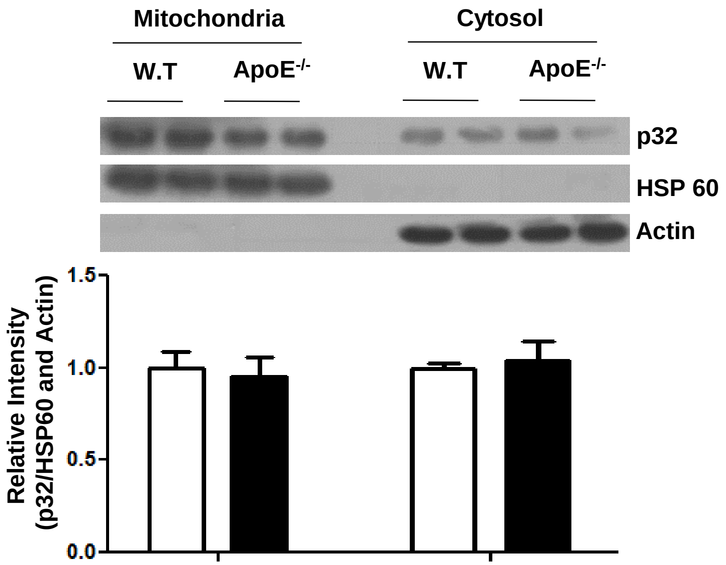

Supplemental Fig. 3. p32m was not changed in aortas of ApoE$^{-/-}$ mice fed an ND. p32 expression in aortic vessels from age-matched (10-week old) WT and ApoE$^{-/-}$ mice fed an ND was not different. n=3.

Suppl. Fig. 4.

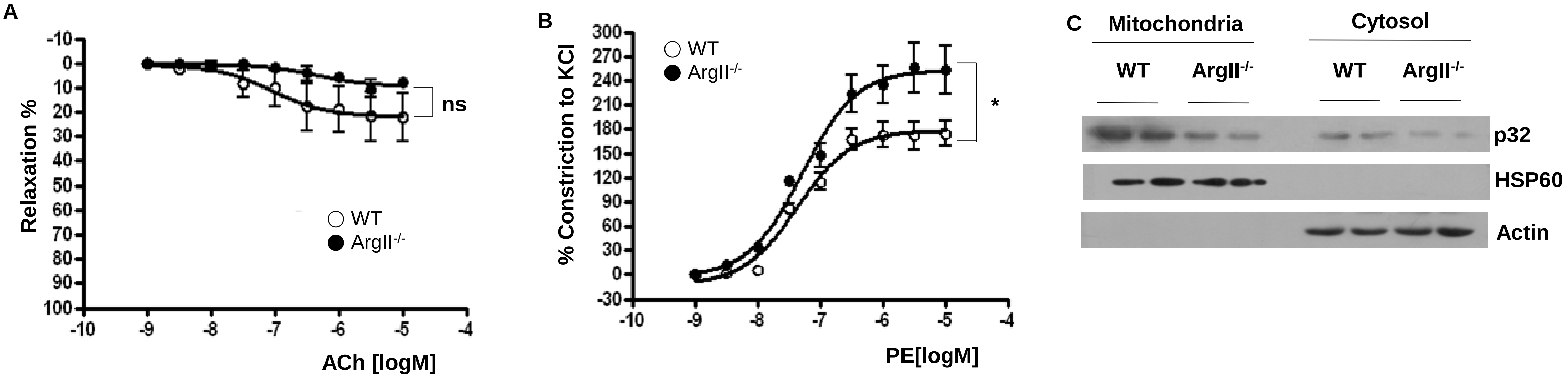

Supplemental Fig. 4. Vasoconstriction responses to PE were enhanced in de-endothelialized aorta (A) from ArgII$^{-/-}$ mice (B) because of the decreased p32m levels (C). * $p<0.05$.
